# The Genetic Landscape of Inherited Retinal Diseases in a Mexican Cohort: Genes, Mutations and Phenotypes

**DOI:** 10.3390/genes12111824

**Published:** 2021-11-19

**Authors:** Cristina Villanueva-Mendoza, Miquel Tuson, David Apam-Garduño, Marta de Castro-Miró, Raul Tonda, Jean Remi Trotta, Gemma Marfany, Rebeca Valero, Vianney Cortés-González, Roser Gonzàlez-Duarte

**Affiliations:** 1 Asociación para Evitar la Ceguera en México, Mexico City 04030, Mexico; villanuevacristina@hotmail.com (C.V.-M.); david.apam@gmail.com (D.A.-G.); 2 DBGen Ocular Genomics, 08028 Barcelona, Spain; mtuson@dbgen.com (M.T.); mart.dcm@gmail.com (M.d.C.-M.); gmarfany@ub.edu (G.M.); 3 CNAG-CRG, Centre for Genomic Regulation (CRG), The Barcelona Institute of Science and Technology, 08036 Barcelona, Spain; raul.tonda@cnag.crg.cat (R.T.); jeanremi.trotta@cnag.crg.eu (J.R.T.); 4 Department of Genetics, Microbiology and Statistics, Faculty of Biology, University of Barcelona, 08028 Barcelona, Spain; 5 Centro de Investigación Biomédica en Red en Enfermedades Raras (CIBERER), Instituto de Salud Carlos III, 08028 Barcelona, Spain

**Keywords:** inherited retinal dystrophies, genetic diagnosis, whole-exome sequencing (WES), *RAB28*, *CWC27*

## Abstract

In this work, we aimed to provide the genetic diagnosis of a large cohort of patients affected with inherited retinal dystrophies (IRDs) from Mexico. Our data add valuable information to the genetic portrait in rare ocular diseases of Mesoamerican populations, which are mostly under-represented in genetic studies. A cohort of 144 unrelated probands with a clinical diagnosis of IRD were analyzed by next-generation sequencing using target gene panels (overall including 346 genes and 65 intronic sequences). Four unsolved cases were analyzed by whole-exome sequencing (WES). The pathogenicity of new variants was assessed by in silico prediction algorithms and classified following the American College of Medical Genetics and Genomics (ACMG) guidelines. Pathogenic or likely pathogenic variants were identified in 105 probands, with a final diagnostic yield of 72.9%; 17 cases (11.8%) were partially solved. Eighteen patients were clinically reclassified after a genetic diagnostic test (17.1%). In our Mexican cohort, mutations in 48 genes were found, with *ABCA4*, *CRB1, RPGR* and *USH2A* as the major contributors. Notably, over 50 new putatively pathogenic variants were identified. Our data highlight cases with relevant clinical and genetic features due to mutations in the *RAB28* and *CWC27* genes, enrich the novel mutation repertoire and expand the IRD landscape of the Mexican population.

## 1. Introduction

Inherited retinal dystrophies (IRDs) comprise a group of diseases with clinical and genetic heterogeneity. According to the Retinal Information Network, over 270 genes are associated with IRDs, which can be inherited as autosomal-recessive (AR), autosomal dominant (AD), X-linked (XL) and mitochondrial conditions. IRDs have an incidence of 1 in 2000–3000 individuals [1] and are one of the leading causes of irreversible blindness in children and young individuals, with a high impact on quality of life and healthcare resources [2].

IRDs are generally characterized by the degeneration of photoreceptors (the specialized and light-sensitive neurons in the retina) and retinal pigment epithelium cells (RPE), although some involve photoreceptor dysfunction, all leading to a significant or total loss of vision. Depending on the cells primarily affected and the course of the disease, IRDs can be classified into specific diagnostic subgroups. The most prevalent form is retinitis pigmentosa (RP), a cone-rod dystrophy that often leads to legal blindness and occupational disability. An uncommon IRD form associated with defective rod photoreceptor functions is congenital stationary night blindness (CSNB). Other forms comprise diseases that predominantly affect the central retina, such as Stargardt’s disease (STGD), cone dystrophy (CD), cone-rod dystrophy (CRD) and achromatopsia (ACHR), a rare congenital cone photoreceptor disorder, with total or partial color blindness being the main clinical trait [1,3]. Infantile forms such as Leber congenital amaurosis (LCA) and early-onset retinal dystrophy (EORD) are considered as severe ocular disorders.

There is a wide spectrum of severity in IRDs, with inter/intrafamilial variability, e.g., family members with the same causative mutation(s) can show different phenotypes. Most IRDs affect only the retina, although additional tissues/organs can be involved in syndromic conditions. A considerable percentage of RP patients (20–30%) present syndromic forms, with Usher (USH), Bardet-Biedl (BBS), Alström, Joubert, Meckel and Senior-Løken syndromes, and Batten disease being the most common forms [4,5]. Each IRD subtype displays a particular phenotype with overlapping clinical traits and different time onsets, hampering an accurate clinical diagnosis without a genetic test [6,7,8].

Due to the significant clinical and genetic heterogeneity of IRDs, the molecular diagnosis of this group of diseases is complex and requires sophisticated sequencing and bioinformatics approaches. During the last three decades, researchers identified nearly 300 IRD genes (RETNET, https://sph.uth.edu/retnet/, accessed on 1 October, 2021) and thousands of causative mutations (ClinVar, https://www.ncbi.nlm.nih.gov/clinvar/, accessed on 1 October 2021). Mutations in IRD genes are frequent among healthy populations: a population study on the whole-exome and -genome sequencing of control cohorts revealed that at least 2.7 billion people worldwide (36% of the population) are carriers of at least one pathogenic IRD recessive allele [9]. Moreover, most patients carry novel pathogenic variants, whose identification relies on either familial studies or data from small geographic areas with some degree of consanguinity, and/or are derived from a founder effect.

Therefore, the genetic diagnosis of specific rare diseases in geographically circumscribed populations provides new relevant data for understanding genetic variation and its relationship with disease. This study outlines genetic testing by targeted gene sequencing and whole-exome sequencing in a cohort of 144 unrelated Mexican patients with clinically diagnosed IRDs from Asociación para Evitar la Ceguera en México in Mexico City (Mexico). Our data highlight cases with relevant clinical and genetic features due to mutations in the *RAB28* and *CWC27* genes, enrich the novel mutation repertoire and unravel the molecular genetic bases of IRDs in the Mexican population ahead of the upcoming gene therapy trials.

## 2. Materials and Methods

This study comprised a group of 144 unrelated probands with clinical diagnoses of IRD. Patients were studied at Asociación Para Evitar la Ceguera en México (APEC) from 2017 to 2021. The study was approved by the Institutional Review Board and adhered to the tenets of the Declaration of Helsinki. Written informed consent was obtained from all the patients or their parents prior to DNA sample collection. IRD diagnosis was based on visual symptoms and clinical ophthalmological examination, which included: best corrected visual acuity (BCVA), refraction and biomicroscopy. Additionally, when available, a full-field electroretinography (ffERG), according to the International Society for Clinical Electrophysiology of Vision (ISCEV); spectral domain optical coherence tomography (OCT), fundus autofluorescence imaging (AF); and color fundus photography were performed. Based on family history, a clinical geneticist classified each case in pedigrees following either autosomal dominant, autosomal recessive or X-linked inheritance patterns. Patients whose family members were unaffected by IRD were considered as sporadic cases. If other family members were possibly affected but the pedigree was not suggestive of a known inheritance pattern, the case was grouped as unclassified. A clinical geneticist systemically evaluated the patients for the presence or absence of additional phenotypic traits that could be associated with a syndrome. In some cases, a central nervous system image or laboratory tests were requested for confirmation.

Genetic analysis was performed at DBGen Ocular Genomics laboratory in Barcelona (Catalonia, Spain), from 2018 to 2021. With the technology available at the time of study: 125 subjects (86.8%) were analyzed with a custom-made targeted gene-sequencing panel comprising 346 genes and 65 intronic sequences, which included all IRD genes plus genes responsible for other inherited eye conditions (Supplementary Materials); 15 subjects (10.4%) were analyzed with a previous version of the panel, which comprised 150 IRD genes; and 4 subjects (2.8%) were analyzed by whole-exome sequencing using Illumina HiSeq 4000 and NovaSeq 6000 sequencing systems. Reads were mapped to human genome build hg19 using the GEM toolkit, allowing up to 4 mismatches. Alignment files containing only properly paired, uniquely mapped reads without duplicates were processed using Picard to add read groups and to remove duplicates. The Genome Analysis Tool Kit (GATK) was used for local realignment. Variant calling was conducted using SAMtools. Functional annotations were added using SnpEff with the GRCh37.75 database. Variants were annotated with SnpSift using the following databases: Human dbSNP build 137, population frequencies from 1000 Genomes, Exome Variant Server and Genome Aggregation Database (gnomAD). The predicted molecular phenotypic effect of identified variants in IRD genes was analyzed in silico using conservation and deleteriousness predictions from dbNSFP, Likelihood ratio test (LRT), Combined Annotation-Dependent Depletion (CADD), MutationTaster, Polymorphism Phenotyping v2 (PolyPhen-2) and Sorting Intolerant from Tolerant (SIFT) algorithms. After these tests, all relevant candidate variants (pathogenic, likely pathogenic or unknown significance) were validated by Sanger sequencing and the subsequent co-segregation analysis of the available family members. All variants were classified according to the American College of Medical Genetics and Genomics (ACMG) [10].

Solved diagnostic cases were considered and identified in the following scenarios: (a) pathogenic or likely pathogenic biallelic variants in an autosomal recessive IRD gene, (b) a pathogenic or likely pathogenic heterozygous variant in an autosomal dominant gene, or (c) a pathogenic or likely pathogenic X-linked hemizygous variant. Solved cases also included patients with a pathogenic or likely pathogenic variants and a second allele containing either a variant of unknown significance (VUS) or a likely bening/hypomorphic variant in a recessive IRD gene, whenever the clinical phenotype and cosegregation analysis supported this classification. In recessive cases, we considered as partially solved diagnoses those where only one pathogenic variant was found, and the second allele was missing. All the other cases were considered as unsolved.

## 3. Results

A cohort of 144 non-related patients was studied, with sixty females (41.7%) and 84 males (58.3%). According to clinical data, patients were classified as RP (47 cases, 32.6%); LCA and EORD (33 cases, 22.9%); STGD, CRD/CD, ACHR and other non-RP dystrophies (37 cases, 25.7%); and syndromic retinal dystrophy (SRD, 20 cases, 13.9%). We also studied six cases of familial exudative vitreoretinopathy/Norrie disease (FEVR) and one case of bilateral persistent fetal vasculature (PFV), overall representing the remaining 4.9%.

**Figure 1:**
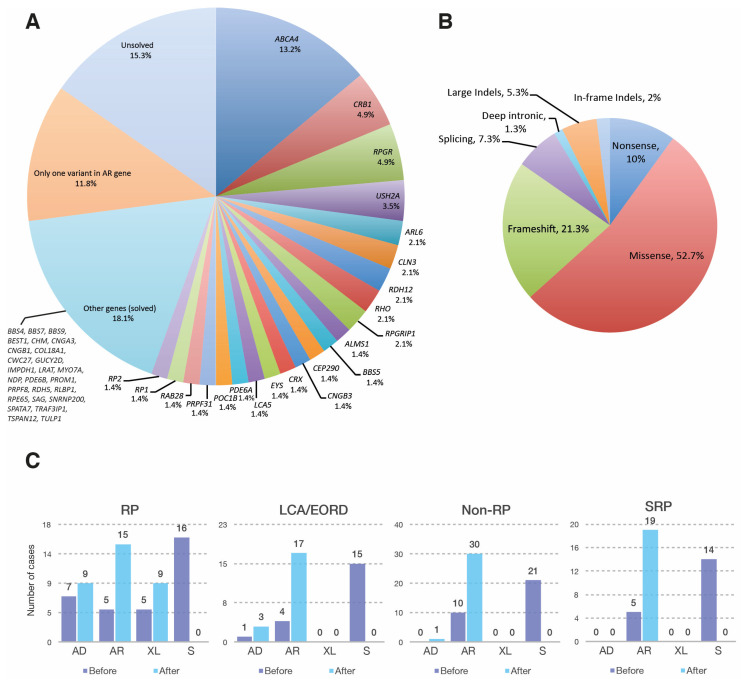
The genetic basis of IRDs in the Mexican APEC cohort. (**A**) Frequency of genes identified in 144 cases with inherited retinal dystrophies; (**B**) Distribution of mutation types in 105 solved cases; (**C**) Classification of inheritance modes in 103 solved cases before (dark blue) and after (light blue) genetic diagnosis. Two cases diagnosed as FEVR/Norrie disease were not classified in the four main phenotypic types, and therefore not included in 1C. AD: autosomal dominant; AR: autosomal recessive; EORD: early onset retinal dystrophy; FEVR: familial exudative vitreoretinopathy; LCA: Leber congenital amaurosis; RP: retinitis pigmentosa; S: sporadic; SRP: syndromic retinal dystrophies; XL: X-linked.

Pathogenic or likely pathogenic variants were identified in 105 probands, with a final diagnostic yield of 72.9% (105/144). Seventeen cases (11.8%) were partially solved, and twenty-two (15.3%) remained unsolved (Figure 1A). The observed clinical phenotypes, following an ophthalmic examination and detailed genetic findings of solved cases, are summarized in Supplementary Table S1. Moreover, over 50 new putatively pathogenic variants were identified (Table 1). Overall, *ABCA4* was the most frequently involved gene (19/105, 18.1%), followed by *RPGR* (7/105, 6.7%) and *CRB1* with (7/105, 6.7%) (Figure 1A). The type of genetic variants identified were mostly missense mutations (52.7%), followed by frameshift (21.3%), nonsense (10%), splicing (7.3%), large indels (5.3%), in frame indels (2%) and deep intronic variants (1.3%) (Figure 1B).

### 3.1. Genetic Landscape of the Mexican IRD Patients

#### 3.1.1. Retinitis Pigmentosa

Initially, the RP group amounted to 47 patients. Mutations in 20 genes were identified in 32 RP patients and one case of choroideremia, with a final genetic diagnostic yield of 70.2% (Table 2). Among the RP genes, *RPGR* was the most frequent (six cases) followed by *RHO* (three cases) (Figure 1A, Table 2). The genetic test results allowed us to assign and/or refine the initial inheritance pattern as inferred from family history, solving 16 RP cases that were initially considered as sporadic (Figure 1C).

#### 3.1.2. Leber Congenital Amaurosis and Early Onset Retinal Dystrophy

Mutations in 11 genes were identified in 20 patients of the LCA/EORD subcohort (33 patients), representing a diagnostic yield of 60.6% (20/33). The most frequently mutated genes were *CRB1* (5 cases), followed by *CEP290, CRX, LCA5, RDH12,* and *RPGRIP1* (2 cases each) (Table 2). Several patients were reclassified after genetic diagnosis: two patients homozygous for novel pathogenic variants in *ALMS1*, and one male patient carrying a novel pathogenic duplication in *RPGR*, c.736_745dupATCCAAGTAG; p.(Ala249AspfsTer37), were classified as Alström syndrome and RP-XL (Table 3), respectively. Although *RPGR* is mostly related to RP, very few reported cases associate specific *RPGR* mutations to LCA/EORD [11].

Moreover, a patient heterozygous for a deletion of exons 7 and 8 and a second pathogenic missense allele, c.494G>A; p.(Gly165Glu), in *CLN3* was reassessed as affected with protracted juvenile neuronal ceroid lipofuscinosis (JNCL) (detailed clinical traits in Table S1). At the time of diagnosis, he did not present any neurologic symptoms although he started suffering seizures at 21 years old.

Based on molecular findings we identified the inheritance pattern in 15 sporadic patients; most of them were autosomal recessive with two de novo AD cases (Figure 1C).

#### 3.1.3. Non-RP Retinal Dystrophies

In this group, patients affected with STGD (twenty cases), CD/CRD (seven cases), ACHR (six cases), CSNB (two cases), Best disease (one case) and unspecified retinal dystrophy (one case) were studied. Pathogenic variants were identified in 12 genes in 31 patients with a final genetic diagnostic yield of 83.7% (31/37). The most frequent gene was *ABCA4* (eighteen cases), followed by *CNGB3* and *POC1B* (two cases each) (Table 2). Four cases were reclassified (Table 3). Two patients had an initial diagnosis of STGD (patients 35 and 48, Table S1). In patient 35, now reclassified as affected with maculopathy, a novel missense putative pathogenic variant was identified in the *CLN3* gene, c.147C>G; p.(Asn49Lys), in homozygosis. At the time of the genetic test, this patient did not show any neurological alteration. Indeed, genetic variants not related to Batten disease were described in this gene [12,13,14]. In patient 48, two pathogenic mutations in *CRB1* were identified and refined the clinical diagnosis from STGD to CRD (Table 3). In reality, one of the identified mutations, c.498_506delAATTGATGG; p.(Ile167_Gly169del), was previously reported to cause macular degeneration in compound heterozygosity [15], in agreement with our results. The last two cases (patients 65 and 94) had an initial diagnosis of ACHR but were reclassified to CRD after identifying the causative mutations in *POC1B* and *RPGRIP1*, respectively.

Based on molecular results, 21 cases initially considered as sporadic were reassigned. Overall, 30 patients presented AR inheritance whereas one case was AD.

#### 3.1.4. Syndromic Retinal Dystrophies

This group initially comprised twenty patients clinically diagnosed with the following syndromes: BBS (six patients), Usher syndrome (four patients), Batten disease (one patient), and Senior-Løken syndrome (one patient). Mutations in 12 genes were identified in 19 patients, with a final genetic diagnostic yield of 95% (19/20). The most frequent gene was *USH2A* (three cases), followed by *ALMS1, ARL6* and *BBS5* (two cases each) (Table 2). A previously undiagnosed patient was found to be homozygous for the *COL18A1* pathogenic variant, c.3523_3524delCT; p.(Leu1175ValfsTer72), associated with Knobloch syndrome (Table S1). The patient’s clinical records allowed us to confirm that the patient had an ocular phenotype associated with this disease, consistent with a diminished visual acuity since early childhood, nystagmus, high myopia, loss of central vision, bilateral cortical opacity, choroidal fundus and macular atrophy. He did not present occipital encephalocele, in agreement with other reported Knobloch syndrome cases [16]. Another patient (70) initially diagnosed as SRP, was reclassified as a CRD related to a novel variant in the *RAB28* gene (see below). Following genetic testing, all sporadic cases (14) were identified as autosomal recessive (Figure 1C).

#### 3.1.5. Other Retinal Dystrophies

Concerning other IRD entities, six patients were affected with familial exudative vitreoretinopathy (FEVR)/Norrie disease and one with bilateral persistent fetal vasculature. Of those, only in two cases causative genetic variants were identified in *TSPAN12* and *NDP* (Table 2).

### 3.2. Novel Genetic Findings in Rare IRD Cases

#### 3.2.1. CWC27

**Figure 2:**
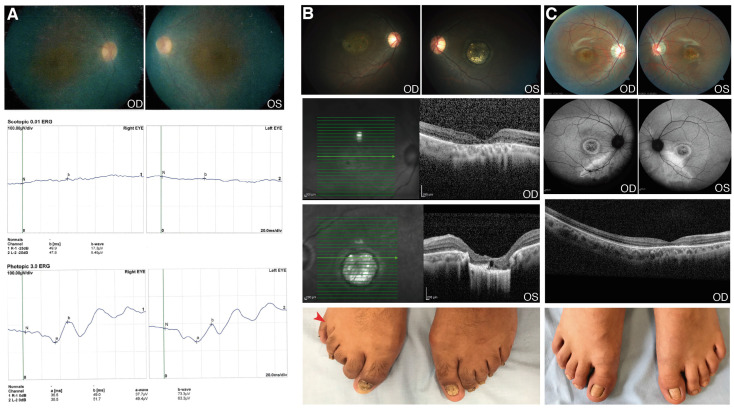
Clinical features of patients with *CWC27* and *RAB28* mutations. (**A**) Patient 51. Fundus photograph and full field electroretinography (ffERG) images (OU). Pale optic disc, attenuated retinal blood vessels and atrophy of retinal pigment epithelium around the vascular arcades. The ffERG demonstrated no response of rods and reduced response of cones. These images were taken when the patient was 16 years old; (**B**) Patient 70. Fundus photograph and optical coherence tomography (OCT) images (OU): Temporal pallor of the optic disc, attenuated retinal arteries and retinal pigment epithelium atrophy in OD. Pale optic disc, colobomatous-like lesion in the macula with hyperpigmented edges; sclera visible and choroidal vessels in OS. The OCT demonstrated macular thinning with atrophy and loss of the outer layers of the entire central zone in OD; colobomatous-like lesion, chorioretinal atrophy and loss of outer layers and choroid in OS. Postaxial polydactyly in right foot (red arrow); (**C**) Patient 71. Fundus photograph, fundus autofluorescence and OCT images (OU). Temporal pallor of the optic disc, mild peripapillary atrophy, hypopigmented lesion in the macula area in OD. Peripapillary atrophy, attenuated retinal blood vessels and hyperpigmented lesion in central area in OS. AF showing macula with central hypoautofluorescence, a ring surrounded by hyperautofluorescence and external hypoautofluorescence; atrophy of retinal pigment epithelium outside the arcades. The OCT demonstrated loss of photoreceptor layer in the central area with pigment epithelium atrophy. Normal feet. OD: oculus dexter (right eye); OS: oculus sinister (left eye); OU: oculus uterque (both eyes).

A 24-year-old male (patient 51) has presented with nystagmus, low vision and nyctalopia since he was an infant (Table S1). Later, he noticed visual field loss. At the time of the clinical examination, the ocular phenotype was RP with a high myopic refractive error: OD -10.00 = 1.00 × 130° and OS -10.00 = 1.50 × 155°. BCVA was +1.4 OU (LogMAR); there was subcapsular lens opacity, vitreous with pigmented cells and generalized bone spicules. Macular OCT showed atrophy of photoreceptors and the ffERG showed no response of rods and a reduced response of cones, with no signs of other associated abnormalities (Figure 2A). Interestingly, this patient was homozygous for a novel 4-nucleotide deletion in *CWC27*, c.1066_1070delGCTGT; p.(Ala356CysfsTer11). Mutations in this gene, which encodes a spliceosome component, were previously reported in rare cases of retinal degeneration with or without skeletal developmental anomalies (Table 4) [17].

#### 3.2.2. RAB28

Patient 70 was a 22-year-old male with diminished visual acuity lasting since childhood, accompanied by photophobia, dyschromatopsia and central vision loss. BCVA was +0.9 OD; +1.0 OS (LogMAR); fundoscopy revealed a bilateral atrophic macula and, in OS, a colobomatous-like lesion in the macula was found. OCT showed a severe atrophy of external layers of the macula and confirmed the macular lesion in OS (Figure 2B). The ffERG showed a diminished response of the rods and cones. The patient also showed unilateral foot post-axial polydactyly. The genetic findings (a novel homozygous variant, c.202G>C; p.(Asp68His), in *RAB28*) allowed for the reassignment of a rare CRD clinical entity (Table 3). *RAB28* was associated with CRD (Table 4), in turn associated with polydactyly in a few cases [18]. Notably, there was another case in our cohort (patient 71) carrying the same mutation in *RAB28*, also in homozygosis, but without polydactyly (Figure 2C). Patient 71 had BCVA +0.7 OD; +1.3 OS. Fundoscopy revealed macular changes, as shown in Figure 1C; OCT revealed a loss of the photoreceptors layer in the central area with atrophy of the retinal pigment epithelium. The ffERG showed a severe reduced cone response and reduced rod response. As summarized in Table S1, the X-rays from hands and feet were normal. Both patients shared the retinal phenotype (photophobia, dyschromatopsia, maculopathy, and atrophy of macular photoreceptors, as detected by OCT), consistent with cone-rod dystrophy.

## 4. Discussion

In this work, we report the genetic analysis of 144 unrelated probands with aclinical diagnosis of IRD, with a solving rate of 72.9%. The highest diagnostic yield was achieved in STGD patients with 95%, whereas other IRDs showed a variable yield, being the lowest in patients with FEVR/Norrie disease (28%). The fact that STGD is mainly caused by mutations in a single gene (*ABCA4*), which is fully covered (exons plus introns) in our gene panel, may account for this high success rate, whereas identifying the genetic cause in highly heterogeneous IRDs becomes more complex.

In our Mexican cohort, mutations in 48 genes were causative of IRDs, with *ABCA4*, *CRB1, RPGR* and *USH2A* as the major contributors. These results are comparable to those previously reported in another Mexican cohort [14], except for *RPGR*, which is much more relevant in our study. Indeed, the repetitive, purine-rich ORF15 exon extremely hampers NGS-based genetic diagnostic approaches. Our approach implemented specific enrichment for this region, allowing us to unambiguously identify 5 out of 7 *RPGR* mutations located at ORF15, which might have been undetected otherwise. On the other hand, genetic studies in populations of different geographic origins identified other major genes, e.g., *EYS* is the main RP gene (up to 20–30% of cases) in some East Asian populations [24,25], likely indicating different genetic structures in these cohorts.

One of the most relevant outcomes of genetic diagnosis is to confirm clinical diagnosis, thus providing an accurate prognosis of disease progression, relevant for clinical management. For instance, patients that are affected by achromatopsia (ACHR) or cone-rod dystrophy (CRD) have similar clinical symptoms such as diminished central vision, photophobia and dyschromatopsia; consequently, it is possible to misdiagnose the two entities. Indeed, securing the diagnosis becomes relevant as the prognosis is different, being better for patients with ACHR [26]. In our cohort, two patients were reclassified from ACHR to CRD. It is worth mentioning that considering the rare entities involving photoreceptor dysfunction (ACHR and CSNB), in the Mexican cohort we confirmed three cases with ACHR and one with CSNB. Overall, around 17% of cases were reclassified after the molecular result in this study (Table 3). Of note, two pediatric patients initially diagnosed as Leber congenital amaurosis (LCA) were reclassified as being affected by Alström syndrome, a multisystemic disease coursing with cone-rod dystrophy. All of the affected children of this syndrome exhibit low vision within the first year of life and, although obesity is also an early and consistent feature, this may be overlooked. Other symptoms, such as hearing loss, develop gradually and are therefore noticed later [27,28,29]. After the results of the molecular test, these two patients were clinically re-evaluated and showed obesity and acanthosis, thus reinforcing the newly assigned clinical entity.

An NGS-based genetic diagnosis also unveils allelic heterogeneity and genotype/phenotype correlations in genes involved in both syndromic and non-syndromic pathologies. *CLN3* is mostly related to Batten disease or juvenile, neuronal ceroid lipofuscinosis (JNCL), but hypomorphic alleles are also associated with non-syndromic RP [12,13,14]. In this study we identified three patients carrying *CLN3* genetic variants showing distinct phenotypes (Table S1). One patient carried the previously reported exon 7–8 deletion in homozygosity and presented the canonical traits associated with Batten disease. A second patient was heterozygote for the same deletion combined with a reported milder missense mutation c.494G>A; p.(Gly165Glu) in trans and presented protracted JNCL. The last patient, affected with retinal degeneration and no other neurological traits at the time of the genetic test, carried a novel missense variant c.147C>G; p.(Asn49Lys) in homozygosity. This missense variant was considered pathogenic by in silico predictions, and is most likely a hypomorphic allele retaining some function. Further studies are needed to confirm these genotype/phenotype correlations.

Two other cases deserve further consideration. First, one patient carried a previously unreported pathogenic homozygous variant in *CWC27*, c.1006_1070delGCTGT; p.(Ala356CysfsTer11). This deletion encompasses five nucleotides in exon 11 and causes a frame-shift that introduces a premature stop codon. The CWC27 protein (472 aa) belongs to the family of cyclophilins of peptidyl-prolyl cis-trans isomerases (PPIases) associated with spliceosome complexes, which most likely function as molecular chaperones during the assembly of spliceosome components [17,30]. Phenotypes associated with mutations in spliceosome-related genes are grouped into two main classes, either syndromic phenotypes with craniofacial and skeletal developmental abnormalities or non-syndromic RP. *CWC27* is the first spliceosome gene associated with a wide spectrum of clinical alterations combining the two different phenotypes, which suggests that *CWC27* is involved in both the early development and maintenance of mature tissue homeostasis [17]. Truncating the mutations that affect the N-terminal protein domain causes a more severe syndromic phenotype, whereas mutations at the C-terminal region still allow the production of a protein with a residual function, compatible with a non-syndromic RP phenotype (see a list of reported mutations in Table 4). The mutation identified in patient 51 is located in the exon 11, well within the C terminal region. Furthermore, a mouse model bearing a homozygous frameshift/truncating mutation in exon 11 [17] showed a very similar phenotype (only ocular, albeit severe) to that of our patient. Therefore, our genetic testing results increase the mutation landscape for *CWC27*, confirm the differential phenotypic effect depending on the altered protein domain, and reinforce the dual role in the development and tissue homeostasis of this gene.

The second gene that deserves deeper discussion is *RAB28.* Notably, two patients (apparently genetically unrelated) homozygous for the same novel missense variant, c.202G>C; p.(Asp68His), displayed a different phenotype. One patient was initially diagnosed as affected with syndromic retinal dystrophy, since he showed unilateral postaxial foot polydactyly (one additional toe) in addition to CRD. A second patient carrying the same homozygous variant was affected only with CRD, without polydactyly. This genetic variant causes an amino acid substitution in a highly conserved nucleotide-binding domain and is predicted as pathogenic by in silico programs (LRT, CADD, MutationTaster, PolyPhen2 and SIFT, among others). Yet, it is classified as VUS according to the ACMG, as no functional assays are available to support its pathogenicity. *RAB28* encodes a member of the Rab subfamily of the RAS-related, small GTPases, which function as major regulators of membrane vesicular trafficking processes [31,32,33]. In the retina, *RAB28* is expressed in the retinal pigment epithelium and photoreceptors. The protein localizes to the basal body and ciliary rootlet and is involved in cone outer segment disk shedding [34]. Very few cases with mutations in *RAB28* were reported (Table 4). Although initially associated with non-syndromic CRD [21,22,23], very recently, postaxial polydactyly, in addition to CRD, was also described in two brothers carrying a missense mutation in *RAB28* [18]. The authors propose that RAB28 associates with the BBSome and is involved in the intraflagellar transport in the cilia. In our study, aside from polydactyly, the ocular phenotype of the patients is highly consistent with these previous reports. This apparent discrepancy in the polydactyly trait can only be reconciled when considering the contribution of modifier genes, which, on the other hand, agrees with the incomplete penetrance and variable expressivity frequently associated with the polydactyly phenotype.

Overall, our work contributes to the genetic portrait of rare ocular diseases of Mesoamerican populations, which are mostly under-represented in genetic studies. Indeed, to attain a full mutational landscape of IRD genes on a global level, genetic studies including different populations are required to fill the gaps in genetic information. Moreover, improved/refined sequencing techniques must be implemented to increase the yield and provide an accurate genetic diagnosis, which is the basis of precision medicine. Finally, we believe that our results involving the mutations in *CWC27* and *RAB28* are relevant to the clinical genetics community as very few mutations were previously reported in these genes. Our findings widen the spectrum of phenotypes, genes and mutations associated with retinal dystrophies, contribute to increasing the diagnostic rate in other laboratories and expand the basic knowledge of IRDs.

## Figures and Tables

**Table 1:** Newly identified putative pathogenic variants.

Case	Gene	NM Number	Chr	HGVS DNA	HGVS Protein Change	Zyg	Inh	ACMG
5	*ABCA4*	NM_000350.3	1	c.3415T>G	p.(Tyr1139Asp)	Het	AR	VUS
19	*ABCA4*	NM_000350.3	1	c.6299G>A; c.6308C>A	p.(Gly2100Glu); p.(Pro2103His)	Het	AR	LPV; LPV
21	*ALMS1*	NM_015120.4	2	c.6828C>A	p.(Cys2276Ter)	Hom	AR	LPV
20	*ALMS1*	NM_015120.4	2	c.7881_7882insGACA	p.(Leu2628AspfsTer33)	Hom	AR	LPV
23	*ARL6*	NM_177976.3	3	c.482C>T	p.(Ala161Val)	Het	AR	VUS
25	*BBS4*	NM_033028.5	15	c.187C>T	p.(Gln63Ter)	Hom	AR	PV
26	*BBS5*	NM_152384.3	2	c.143-1G>C		Hom	AR	PV
27	*BBS5*	NM_152384.3	2	c.143-1G>C		Het	AR	PV
27	*BBS5*	NM_152384.3	2	c.559_560insGA	p.(Ile187ArgfsTer8)	Het	AR	PV
28	*BBS7*	NM_176824.3	4	c.302T>A	p.(Leu101His)	Hom	AR	VUS
35	*CLN3*	NM_001042432.2	16	c.147C>G	p.(Asn49Lys)	Hom	AR	VUS
40	*CNGB3*	NM_019098.5	8	c.701G>A	p.(Cys234Tyr)	Het	AR	VUS
42	*CRB1*	NM_201253.3	1	c.2630_2631dup	p.(Leu878PhefsTer5)	Het	AR	PV
43	*CRB1*	NM_201253.3	1	c.3166G>T	p.(Asp1056Tyr)	Hom	AR	LPV
45	*CRB1*	NM_201253.3	1	c.3881G>A	p.(Cys1294Tyr)	Het	AR	LPV
46	*CRB1*	NM_201253.3	1	c.3884_3904del	p.(Glu1295_Cys1301del)	Het	AR	LPV
47	*CRB1*	NM_201253.3	1	Deletion of exons 8-9 and insertion of exon 6 (inverted)		Hom	AR	PV
50	*CRX*	NM_000554.6	19	c.564del	p.(Ala189ProfsTer5)	Het	AD	PV
51	*CWC27*	NM_005869.4	5	c.1066_1070del	p.(Ala356CysfsTer11)	Hom	AR	PV
53	*EYS*	NM_001142800.2	6	c.2287T>G	p.(Trp763Gly)	Het	AR	VUS
53	*EYS*	NM_001142800.2	6	c.8606C>G	p.(Ser2869Ter)	Het	AR	PV
52	*EYS*	NM_001142800.2	6	Duplication of exons 4-5		Het	AR	PV
54	*GUCY2D*	NM_000180.4	17	c.1773del	p.(Asn591LysfsTer46)	Het	AR	PV
55	*IMPDH1*	NM_000883.4	7	c.940A>G	p.(Lys314Glu)	Het	AD	VUS
56	*LCA5*	NM_001122769.3	6	c.66_72delTTACTTins302nt		Hom	AR	PV
57	*LCA5*	NM_001122769.3	6	c.1368dup	p.(Glu457ArgfsTer14)	Hom	AR	PV
58	*LRAT*	NM_004744.5	4	c.224C>T	p.(Pro75Leu)	Het	AR	LPV
58	*LRAT*	NM_004744.5	4	c.504C>A	p.(Cys168Ter)	Het	AR	PV
59	*MYO7A*	NM_000260.4	11	c.767A>G	p.(Tyr256Cys)	Het	AR	LPV
59	*MYO7A*	NM_000260.4	11	c.6071G>C	p.(Arg2024Pro)	Het	AR	LPV
60	*NDP*	NM_000266.4	X	c.355A>C	p.(Thr119Pro)	Hem	XL	LPV
61	*PDE6A*	NM_000440.3	5	c.2380_2382del	p.(Glu794del)	Het	AR	LPV
63	*PDE6B*	NM_000283.4	4	c.1682A>G	p.(His561Arg)	Hom	AR	LPV
65	*POC1B*	NM_172240.3	12	c.144del	p.(Lys48AsnfsTer16)	Het	AR	PV
67	*PRPF31*	NM_015629.4	19	c.176del	p.(Met59SerfsTer6)	Het	AD	PV
70, 71	*RAB28*	NM_001017979.3	4	c.202G>C	p.(Asp68His)	Hom	AR	VUS
72	*RDH12*	NM_152443.3	14	c.529G>C	p.(Ala177Pro)	Hom	AR	VUS
79	*RLBP1*	NM_000326.5	15	Deletion of exon 6		Hom	AR	PV
81	*RP1*	NM_006269.2	8	c.4709del	p.(Gly1570GlufsTer10)	Het	AR	LPV
83	*RP2*	NM_006915.3	X	c.524A>C	p.(His175Pro)	Hem	XL	LPV
87	*RPGR*	NM_001034853.2	X	c.736_745dup	p.(Ala249AspfsTer37)	Hem	XL	PV
88	*RPGR*	NM_001034853.2	X	c.1481G>T	p.(Gly494Val)	Hem	XL	VUS
89	*RPGR*	NM_001034853.2	X	c.2455_2468dup		Hem	XL	VUS
90	*RPGR*	NM_001034853.2	X	c.2543del	p.(Glu848GlyfsTer241)	Hem	XL	PV
91	*RPGR*	NM_001034853.2	X	c.2587G>T	p.(Glu863Ter)	Hem	XL	VUS
93	*RPGRIP1*	NM_020366.4	14	c.2988del	p.(Glu996AspfsTer5)	Het	AR	LPV
93	*RPGRIP1*	NM_020366.4	14	Deletion of exons 2–17		Het	AR	PV
98	*TRAF3IP1*	NM_015650.4	2	c.88C>T	p.(Pro30Ser)	Hom	AR	VUS
99	*TSPAN12*	NM_012338.4	7	c.301dup	p.(Leu101ProfsTer16)	Het	AD	PV
102	*USH2A*	NM_206933.4	1	c.9473del	p.(Lys3158SerfsTer2)	Het	AR	PV
105	*USH2A*	NM_206933.4	1	c.8126_8127dupCA	p.(Asn2710GlnfsTer7)	Hom	AR	PV

ACMG: American College of Medical Genetics and Genomics classification; AD: autosomal dominant; AR: autosomal recessive; Chr: chromosome; Het: heterozygous; Hem: hemizygous; HGVS; Human Genome Variation Society (nomenclature); Hom: homozygous; Inh: inheritance; LPV: likely pathogenic variant; PV: pathogenic variant; VUS: variant of unknown significance; XL: X-linked; Zyg: zygosity.

**Table 2:** Number of patients with positive genetic testing per IRD.

IRD	Patients	Genes Identified
**Non-syndromic**		
Achromatopsia	3	*CNGA3* (1-AR), *CNGB3* (2-AR)
Best disease	1	*BEST1* (1-AD)
Choroideremia	1	*CHM* (1-XL)
Cone-rod dystrophy/cone dystrophy	8	*ABCA4* (1-AR), *ARL6* (1-AR), *CRB1* (1-AR), *POC1B* (2-AR), *PROM1* (1-AR), *RAB28* (1-AR), *RPGRIP1* (1-AR)
Congenital stationary night blindness	1	*RDH5* (1-AR)
Early-onset retinal dystrophy	6	*CEP290* (1-AR)*, CRB1* (1-AR)*, CWC27* (1-AR)*, GUCY2D* (1-AR)*, RDH12* (2-AR)
Familial exudative vitreoretinopathy	1	*TSPAN12* (1-AD)
Leber congenital amaurosis	14	*CEP290* (1-AR), ***CRB1*** (4-AR), *CRX* (2-AD), *IMPDH1* (1-AD), *LCA5* (2-AR), *RPE65* (1-AR), *RPGRIP1* (2-AR), *SPATA7* (1-AR)
Maculopathy, retinal degeneration	1	*CLN3* (1-AR)
Norrie disease	1	*NDP* (1-XL)
Retinitis pigmentosa	32	*ABCA4* (1-AR), *CNGB1* (1-AR), *CRB1* (1-AR), *EYS* (2-AR), *LRAT* (1-AR), *PDE6A* (2-AR), *PDE6B* (1-AR), *PRPF31* (2-AD), *PRPF8* (1-AD), *RDH12* (1-AR), ***RHO*** (3-AD), *RLBP1* (1-AR), *RP1* (1-AD, 1-AR), *RP2* (2-XL), ***RPGR*** (7-XL), *SAG* (1-AD), *SNRNP200* (1-AD), *TULP1* (1-AR), *USH2A* (1-AR)
Stargardt disease	17	***ABCA4*** (17-AR)
**Syndromic**		
Alström syndrome	2	*ALMS1* (2-AR)
Bardet-Biedl syndrome	7	*ARL6* (2-AR), *BBS4* (1-AR), *BBS5* (2-AR), *BBS7* (1-AR), *BBS9* (1-AR)
Con-rod dystrophy, syndromic	1	*RAB28* (1-AR)
Batten disease/JNCL	2	*CLN3* (2-AR)
Knobloch syndrome	1	*COL18A1* (1-AR)
Senior-Løken syndrome	1	*TRAF3IP1* (1-AR)
Usher syndrome	5	***USH2A*** (4-AR), *MYO7A* (1-AR)
Total	105	

AD: autosomal dominant; AR: autosomal recessive; IRD: inherited retinal dystrophy; JNCL: juvenile neuronal ceroid lipofuscinosis; XL: X-linked. In bold, major genes in each class (with 3 or more cases identified).

**Table 3:** Reclassified cases.

Case	Initial Diagnosis	Gene	Final Diagnosis
3	Maculopathy	*ABCA4*	STGD
35	STGD	*CLN3*	Maculopathy and RD
48	STGD	*CRB1*	CRD
43	RP vs. STGD	*CRB1*	RP
33	RP	*CHM*	Choroideremia
103	RP	*USH2A*	Usher II
38	ACHR vs. BCM	*CNGA3*	ACHR
65	ACHR	*POC1B*	CRD
94	ACHR	*RPGRIP1*	CRD
20	LCA	*ALMS1*	Alström syndrome
21	LCA	*ALMS1*	Alström syndrome
87	EORD	*RPGR*	XLRP
51	EORD vs. XLRP	*CWC27*	EORD
36	EORD	*CLN3*	Protracted JNCL
28	BBS vs. Alström	*BBS7*	BBS
41	SRP	*COL18A*	Knobloch syndrome
70	SRP	*RAB28*	CRD + polydactyly
104	Usher II vs. Usher III	*USH2A*	Usher II

ACHR: achromatopsia; BBS: Bardet-Biedl syndrome; BCM: blue cone monochromatism; CRD: cone-rod dystrophy; EORD: early onset retinal dystrophy; LCA: Leber congenital amaurosis; RD: retinal degeneration; RP: retinitis pigmentosa; SRP: syndromic retinitis pigmentosa; STGD: Stargardt disease; XLRP: X-linked retinitis pigmentosa.

**Table 4:** Mutations associated with IRDs in the *CWC27* and *RAB28* genes.

Gene	HGVS DNA	HGVS Protein Change	Phe	Zyg	Ref
*CWC27*	c.19C>T	p.(Gln7Ter)	RP, syndromic	Het	[17]
	c.355C>T	p.(Arg119Ter)	RP, syndromic	Hom	[19]
	c.427C>T	p.(Arg143Ter)	RP, syndromic	Het	[17]
	c.495G>A	p.(Leu167GlyfsTer3)	RP, syndromic	Hom	[17]
	c.599+1G>A	p.[Val166LysfsTer3; Val191LysfsTer3]	RP, syndromic	Hom	[17]
	c.617C>	p.(Ser206Ter)	RD	Het	[17]
	c.943G>T	p.(Glu315Ter)	RP, syndromic	Hom	[17]
	c.1002dupA	p.(Val335SerfsTer13)	RP	Het	[17]
	**c.1066_1070delGCTGT**	**p.(Ala356CysfsTer11)**	**EORD**	**Hom**	**This study**
*RAB28*	c.37del	p.(Leu13Ter)	CRD (AR)	Hom	[20]
	c.55G>A	p.(Gly19Arg)	CRD (AR), PAP, and myopia	Hom	[18]
	c.68C>T	p.(Ser23Phe)	CRD (AR)	Hom	[21]
	c.76−9A>G	p.(Thr26ValfsTer4)	CRD (AR)	Hom	[20]
	c.77C>A	p.(Thr26Asn)	CRD (AR)	Het	[20]
	c.172+1G>C		CRD (AR)	Hom	[22]
	**c.202G>C**	**p.(Asp68His)**	**CRD (AR) with or without unilateral PAP**	**Hom**	**This study**
	c.321G>A	p.(Trp107Ter)	CRD (AR)	Hom	[20]
	c.409C>T	p.(Arg137Ter)	CRD (AR)	Hom/Het	[20,23]
	c.565C>T	p.(Gln189Ter)	CRD (AR)	Hom	[23]
	c.651T>G	p.(Cys217Trp)	CRD (AR)	Hom	[22]

CRD (AR): cone-rod dystrophy (autosomal recessive); EORD: early onset retinal dystrophy; Het: heterozygous; Hem: hemizygous; HGVS; Human Genome Variation Society (nomenclature); Hom: homozygous; PAP: postaxial polydactyly; Phe: reported phenotype; RD: retinal degeneration; RP: retinitis pigmentosa. In bold, mutations identified in this study.

## Data Availability

The data presented in this study are available on request from the corresponding authors. Novel genetic variants have been submitted to the ClinVar database: https://www.ncbi.nlm.nih.gov/clinvar/, submitted on 14 June 2021.

## Supplementary Materials

The following are available online at https://www.mdpi.com/article/10.3390/genes12111824/s1, Supplementary Materials: IRD genes and genes responsible for other inherited eye conditions analysed. Table S1: Detailed information of solved cases.
