# Supplementary material for: The Genetic Landscape of Inherited Retinal Diseases in a Mexican Cohort: Genes, Mutations and Phenotypes
Source: Genes (Basel). 2021 Nov 19;12(11):1824. doi: 10.3390/genes12111824 (PMC8624043; doi:10.3390/genes12111824)
Supplement: Supplementary file 1 [file genes-12-01824-s001.zip › Supplementary Data.pdf]

genes

Supplementary Material

IRD genes and genes responsible for other inherited eye conditions analysed (coding regions - exons):

ABCA4, ABCC6, ABHD12, ACBD5, ACO2, ADAM9, ADAMTS18, ADIPOR1, AGBL5, AHI1, AHR, AIPL1, ALMS1, ANKS6, AP3B1, ARHGEF18, ARL13B, ARL2BP, ARL3, ARL6, ARSG, ASB10, ASRGL1, ATF6, B9D1, B9D2, BBIP1, BBS1, BBS10, BBS12, BBS2, BBS4, BBS5, BBS7, BBS9, BEST1, BLOC1S3, BLOC1S6, BTD, C10orf11, C12orf65, C1QTNF5, C21orf2, C2orf71, C5orf42, C8orf37, CA4, CABP4, CACNA1F, CACNA2D4, CAPN5, CC2D2A, CDH16, CDH23, CDH3, CDHR1, CEP19, CEP164, CEP250, CEP290, CEP41, CEP78, CEP83, CERKL, CFH, CHM, CIB2, CLCC1, CLN3, CLRN1, CLUAP1, CNGA1, CNGA3, CNGB1, CNGB3, CNNM4, COL11A1, COL11A2, COL18A1, COL2A1, COL4A1, COL4A3, COL4A4, COL4A5, COL9A1, COL9A2, COL9A3, CRB1, CRYAA, CRYBA4, CRX, CSPP1, CTNNA1, CWC27, CYP1B1, CYP4V2, DFNB31, DHDDS, DHX38, DNAJC17, DNM1L, DRAM2, DTHD1, DTNBP1, EDN3, EDNRB, EFEMP1, ELOVL4, EMC1, ESPN, EYS, FAM161A, FLVCR1, FOXC1, FRMD7, FSCN2, FZD4, GDF6, GLIS2, GNAT1, GNAT2, GNB3, GPR125, GPR143, GPR179, GPR98, GRK1, GRM6, GUCA1A, GUCA1B, GUCY2D, GYLTL1B, HARS, HESX1, HGSNAT, HK1, HPS1, HPS3, HPS4, HPS5, HPS6, IDH3A, IDH3B, IFT43, IFT140, IFT172, IFT27, IFT80, IFT81, IMPDH1, IMPG1, IMPG2, INPP5E, INVS, IQCB1, IRX1, JAG1, KCNJ13, KCNV2, KIAA1549, KIF11, KIZ, KLHL7, LCA5, LMX1B, LRAT, LRIT3, LRP5, LTBP2, LZTFL1, MAK, MAPKAPK3, MC1R, MERTK, MFN2, MFRP, MFSD8, MIR204, MITF, MKKS, MKS1, MVK, MYO7A, MYOC, NDP, NEK2, NEK8, NEUROD1, NMNAT1, NPHP1, NPHP3, NPHP4, NR2E3, NR2F1, NRL, NYX, OAT, OCA2, OFD1, OPA1, OPA3, OPN1LW, OPN1MW, OPN1SW, OPTN, OTX2, PANK2, PAX2, PAX3, PAX6, PCDH15, PCYT1A, PDE6A, PDE6B, PDE6C, PDE6D, PDE6G, PDE6H, PDZD7, PEX1, PEX2, PEX7, PHYH, PITPNM3, PITX2, PLA2G5, PLG, PLK4, POC1B, POC5, POLG, POMGNT1, PNPLA6, PRCD, PRDM13, PROKR2, PROM1, PRPF3, PRPF31, PRPF4, PRPF6, PRPF8, PRPH2, PRPS1, RAB28, RAX2, RB1, RBP3, RBP4, RCBTB1, RD3, RDH11, RDH12, RDH5, REEP6, RGR, RGS9, RGS9BP, RHO, RIMS1, RLBP1, ROM1, RP1, RP1L1, RP2, RP9, RPE65, RPGR, RPGRIP1, RPGRIP1L, RS1, RTN4IP1, SAG, SAMD11, SCAPER, SCLT1, SCN2A, SDCCAG8, SEMA4A, SEMA6B, SH3PXD2B, SLC24A1, SLC24A5, SLC25A46, SLC38A8, SLC45A2, SLC4A3, SLC7A14, SNAI2, SNRNP200, SOX10, SOX2, SOX3, SPATA7, SPP2, STRA6, SYT9, TCTN1, TCTN2, TCTN3, TEK, TIMM8A, TIMP3, TMEM107, TMEM126A, TMEM138, TMEM216, TMEM231, TMEM237, TMEM67, TMPRSS4, TOPORS, TPP1, TRAF3IP1, TREX1, TRIM32, TRNT1, TRPM1, TSPAN12, TSPAN6, TTC21B, TTC8, TTLL5, TTPA, TTR, TUB, TUBGCP4, TUBGCP6, TULP1, TYR, TYRP1, UCHL1, UNC119, USH1C, USH1G, USH2A, VCAN, VHL, VPS13B, WDPCP, WDR19, WDR36, WFS1, ZNF408, ZNF423, ZNF513

Non-coding regions analysed (intronic and/or regulatory regions):

ABCA4 (all introns), *CEP290 c.2991+1655A>G, CHM c.315-4587T>A, CHM c.315-1536A>G, COL2A1 c.1527+104T>G, COL2A1 c.1527+135G>A, COL4A5 c.2395+2750A>G, COL4A5 c.385-719G>A, COL4A3 c.4929-388G>T, GPR143 c.659-131T>G, OFD1 c.935+706A>G, OPA1 c.610+360G>A, OPA1 c.610+364G>A, PRPF31 c.1374+654C>G, RB1 c.2490-1398A>G, RPGRIP1 promoter, USH2A c.7595-2144A>G, USH2A c.8845+628C>T, USH2A c.9959-4159A>G, USH2A c.5573-834A>G*
